# Learning through difficult disclosures of cancer progression: experiences of physicians and nurses in training

**DOI:** 10.1186/s12909-026-08688-9

**Published:** 2026-02-06

**Authors:** Julia Kolly, Kristopher Lamore, Louise Haldemann, Benedetta Franceschiello, Virginie Poulin, Sophie Lelorain

**Affiliations:** 1 Psychology Institute, PHASE – Research Centre for Psychology of Health, Aging and Sport Examination, Quartier Centre, University of Lausanne, Lausanne, 1015 Switzerland; 2 CNRS, UMR 9193 - SCALab - Sciences Cognitives et Sciences Affectives, University of Lille, Lille, F 59000 France

**Keywords:** Medical communication, Cancer progression, Psycho-oncology, Thematic analysis, Medical education

## Abstract

**Objective:**

Breaking the news of cancer progression is a complex task that requires a balance between clinical accuracy and relational sensitivity. Although research has examined the perspectives of experienced healthcare professionals, little is known about how trainees navigate these consultations. This study aims to explore the experiences of medical and nursing trainees in disclosing disease progression, focusing on communication strategies, emotional impacts, and professional development.

**Methods:**

Eighteen semi-structured interviews were conducted with trainees in Swiss hospitals (11 oncologists and 7 nurses). The data were analyzed via thematic analysis.

**Results:**

Trainees described disclosure as a carefully prepared, emotionally demanding process requiring clarity, empathy, and teamwork. Five themes emerged, with particular emphasis on experiential learning and the emotional burden of disclosure. Formal training and protocols provided limited guidance, while experience, supervision, and peer support were crucial in developing communication skills. Variability in patient and family reactions influenced decision-making and underscored the need for flexible, relationally attuned communication.

**Conclusion:**

Disclosing cancer progression is a demanding experience for trainees, who described the need to adapt their communication, manage emotional responses, and rely on interprofessional collaboration. Their accounts highlight the central role of experience, supervision, and supportive environments in shaping how they navigate these challenging consultations.

## Introduction

Recent advances in oncological treatments and increased life expectancy have significantly lengthened and complicated cancer care trajectories. Consequently, patients more frequently undergo successive lines of treatment that prolong cancer care, while cancer remains one of the most prevalent and lethal diseases worldwide [1]. Within this context, consultations addressing disease progression due to treatment resistance, alongside the proposal of new therapeutic strategies, are becoming more frequent. It is essential to distinguish progression from recurrence: the former refers to ongoing disease growth despite active treatment, whereas the latter refers to the reappearance of cancer following remission. Progression, often indicative of therapeutic resistance, can arise at various stages of care and across cancer types, necessitating adjustments in treatment strategies [2].

Despite the growing prevalence of these emotionally complex and sensitive consultations, scholarly attention to the communication of progression remains limited [3, 4]. Cancer progression is considered bad news, i.e., information that significantly and negatively alters a patient’s perception of their future [5]. Indeed, it is particularly concerning for two reasons: first, it indicates that current treatments are no longer sufficiently effective, requiring a change in therapy,second, this change prompts a reassessment of prognosis, as the most effective treatments are generally administered first [2].

Delivering bad news is a frequent and essential aspect of oncology practice. Healthcare professionals (HCPs) consistently report these encounters as emotionally challenging, requiring both professional expertise and emotional sensitivity [6]. Literature on breaking bad news in the context of diagnosis, recurrence, and transition to palliative care has revealed the significant psychological burden patients and caregivers (P&Cs) often experience, manifesting as anxiety, depression, hopelessness, uncertainty, and reduced self-efficacy [7–10]. HCPs themselves are not immune to these negative effects: Matthews et al. [4] demonstrated that HCPs experience stress, intrusive thoughts, and compromised well-being following difficult conversations, with repercussions sometimes extending into their personal lives. Physiological symptoms, including heightened arousal during such encounters, have also been documented [11]. Recent findings have suggested that disclosing cancer progression is particularly emotionally taxing and that emotional responses to bad news tend to vary with clinical experience, suggesting that trainees may be particularly vulnerable to negative effects [12, 13]. Indeed, in prior interviews with HCPs, trainees were consistently identified as particularly vulnerable during consultations involving cancer progression [13]. These findings, together with evidence from the literature on the emotional burden of delivering bad news, underscore the need to explore how medical and nursing trainees experience these consultations to inform educational interventions and provide appropriate emotional and pedagogical support.

The aforementioned challenges underscore how trainees perceive and engage with their oncology training. Specializing in oncology is rarely a random choice. Faivre et al. [14] found that the relational dimension of oncology, including intense and diverse patient interactions, is a key motivator for trainees. Nevertheless, delivering unfavorable news remains one of the most challenging aspects of the profession, with trainees often feeling unprepared and emotionally affected [6, 15, 16]. This perceived lack of preparedness is consistent with prior reviews showing that training in breaking bad news is often insufficient, inconsistently implemented, and overly theoretical, with limited opportunities for experiential learning and feedback [17]. Despite the emotional toll, trainees generally recognize the importance of difficult conversations as an integral aspect of their professional role [16, 18].

This study to explore how medical and nursing trainees experience consultations in which a change in treatment is discussed due to cancer progression. Our research question was as follows: *How do medical and nursing trainees experience consultations in which cancer progression is disclosed?* Specifically, we sought to examine the communication strategies employed, the emotional impact of delivering bad news, the support and resources utilized, the influence of family caregivers in these interactions, and how these experiences shape professional identity and learning.

## Methods

### Study design

This qualitative study was grounded in a socio-constructivist paradigm [19] that emphasizes how individuals construct their perceptions of reality through social, cultural, and historical contexts [20, 21].

This study is part of the first author’s doctoral research project. Although this project has led to other publications [13, 22], the present manuscript is based on a distinct qualitative dataset. Specifically, this study analyzes interviews conducted with healthcare professionals in training, whereas Kolly et al. [13] focused on experienced healthcare professionals. While both studies share a common conceptual framework, they address different research questions and professional stages, ensuring the originality of the present contribution.

Semi-structured interviews were selected for their capacity to capture medical and nursing trainees’ perspectives while allowing flexibility in the interview process. This method is well‑suited to exploring complex phenomena [23] and enables the emergence of themes that reflect lived realities. The exploratory nature of the topic further justified this methodological choice. The study was conducted in accordance with the Consolidated Criteria for Reporting Qualitative Research (COREQ) guidelines [24].

### Participants

Medical and nursing trainees were recruited between June 2024 and September 2025. Although the recruitment period extended from June 2024 to September 2025, this duration reflects the challenges of accessing a highly solicited and clinically burdened population. Oncology trainees were often difficult to reach due to heavy clinical workloads and limited availability. In addition, recruitment was initially planned in both Switzerland and France; however, despite extensive outreach efforts, no trainees based in France agreed to participate. These challenges contributed to the extended recruitment period and underscore the difficulty of engaging trainees on this sensitive topic.

Eligible participants were at least 18 years old, enrolled in medical or surgical internships or nursing training programs, engaged in clinical activity involving patients with digestive, dermatological, thoracic, or breast cancer, and participated in consultations to announce cancer progression (CACPs) when at least one treatment was still available. No exclusion criteria were defined. Recruitment concluded when data sufficiency was reached, defined as the point at which additional interviews no longer generated new themes or meaningful variations relevant to the research questions. This was assessed through ongoing, iterative analysis conducted in parallel with data collection, allowing the research team to determine that subsequent interviews largely confirmed existing patterns rather than extending them [25].

The aforementioned cancer types were selected in collaboration with oncologists due to their poor prognoses, marked by aggressive progression and frequent late-stage diagnoses. Thus, treatment resistance and subsequent therapeutic changes are more common in these cases compared with those involving other cancer types [26, 27]. According to clinical experience and current scientific literature, these cancers are considered likely to progress within one year,depending on the type of cancer, 50–70% of patients are expected to die within 12 months [28, 29].

### Procedure

An invitation to participate in the study was emailed to eligible trainees, accompanied by an informational flyer. Snowball sampling was employed; thus, the invitation was further shared with potentially eligible trainees. Interested individuals were encouraged to contact the sender (JK, LH, BF, or VP) for additional details about the study and to obtain an information letter. Interviews were scheduled upon confirmation of participation.

JK, a female PhD candidate in health psychology in the final stages of her PhD, conducted several interviews. JK is trained in semi-structured interviewing and qualitative research methods at the university level; she also trained LH, BF, and VP (Master’s students in psychology) to conduct interviews. All four researchers were involved in data collection. No prior relationship existed between the interviewers and participants. At the start of each interview, the researchers introduced themselves as psychology researchers based in Switzerland studying cancer progression in oncology.

Each interview began with the same initial question: “*When reviewing the exam results, you observe a progression of the disease for your patient who has advanced cancer. How does the consultation to communicate these results unfold, considering that despite the advanced nature of the cancer, there is still at least one possible line of treatment?*” The conversation then explored common themes across both professional groups (medical or nursing trainees), including communication strategies, the emotional impact of delivering bad news, the support and resources used, and the influence of family caregivers during such consultations. Field notes were taken during and after the interviews, which were conducted either via videoconference or in person, depending on participants’ preference. All interviews were audio-recorded using two dictaphones or a videoconferencing platform to ensure data reliability. Transcriptions were completed verbatim, with all identifying information (e.g., names and locations) pseudonymized. Additionally, descriptive data were collected during the interviews, including the participants’ age, gender, profession, years of experience in oncology, and their affiliation with specific cancer units or hospitals.

### Analysis

The interviews were analyzed using Braun and Clarke’s thematic analysis method, following an inductive approach [25, 30]. This approach enables the breakdown of each interview's narrative structure to identify thematic patterns across the entire dataset [31]. NVivo software (NVivo © 2021 QSR International Pty Ltd.) was used to support the analysis. The process followed six phases: (1) familiarization with the data, (2) generation of initial codes (without abstraction at this stage), (3) identification of potential themes, (4) review of themes, (5) definition and naming of themes, and (6) production of the final report, including illustrative verbatim excerpts.

Although the thematic analysis was conducted across the entire dataset without separating professions, particular attention was paid to identifying potential convergences and divergences between medical and nursing trainees during the coding and theme development phases. Where profession-specific roles or experiences emerged (e.g., physicians more frequently leading the disclosure, nurses more often providing emotional support), these distinctions were explicitly reported in the Results section. Overall, the core themes reflected largely shared experiences across both professional groups, which supported the decision to conduct a combined analysis while highlighting profession-related nuances when relevant.

The decision to conduct a single thematic analysis was driven by the study’s aim to explore shared experiences of cancer progression disclosure within interprofessional oncology settings. As these consultations involve closely intertwined medical and nursing roles, a combined analysis allowed us to capture common meaning-making processes while avoiding premature professional segmentation. Professional differences were nonetheless preserved analytically by systematically attending to profession during coding and by explicitly reporting profession-specific roles and experiences when they emerged.

### Ethical approval

This research was conducted in accordance with the protocol, the tenets of the Declaration of Helsinki, the principles of good clinical practice, the Law on Human Research (LRH), the Ordinance on Human Research (ORH), and other relevant local regulations. The study was approved by the Research Ethics Committee of the University of Lausanne (reference n°E_SSP_062022_0000I).

### Informed consent

All participants received comprehensive information about the study’s objectives, procedures, potential risks, and benefits, and provided written informed consent prior to participation. Participation was entirely voluntary, and the participants were free to withdraw from the study at any time without consequence. Informed consent was obtained after the participants had read the information sheet, between June 2024 and October 2025. Privacy and confidentiality were ensured through the pseudonymization of all data.

## Results

### Participant characteristics

The recruitment process involved extensive outreach efforts by the research team, including over 100 emails and 80 phone calls to various institutions, ultimately leading to the identification of 33 potential participants. Of these, 12 did not respond, three declined to participate, and 18 agreed to participate in the interviews. There were no dropouts. There were 11 physician trainees and seven nursing trainees, all working in public hospitals in Switzerland. The interviews lasted between 30 minutes and 1.3 hours, with an average duration of 53.6 minutes.

The participants’ characteristics are presented in Table 1. The mean age was 30.7 years (range: 24–42 years). The gender distribution was particularly unbalanced among nurses (100% female) and physicians (64% female and 36% male). All but three medical trainees worked full-time. The nursing trainees worked at rates ranging from 60% to 100%. The physicians had an average of 4.09 years of oncology experience (range 1–5), and the nurses had an average of 2.67 years (range 1–5).

**Table 1:** Participant characteristics (*N* = 18)

**Characteristic**	***Profession***		***Total***	**%**
	***Oncologist trainees (n = 11)***	***Nurse trainees (n = 7)***		
Age (y)				
<25	0	1	1	6
26–30	4	2	6	33
31–35	3	3	6	33
>35	4	1	5	28
Gender				
Female	7	7	14	78
Male	4	0	4	22
Activity rate				
<70%	0	3	3	17
80%	1	1	2	11
90%	2	0	2	11
100%	8	3	11	61
Number of years of experience in oncology				
<2	2	2	4	22
2–4	5	4	9	50
5–7	3	1	4	22
>7	1	0	1	6

### Themes organization

Given that most medical and nursing trainees have only minimal CACP consultation experience, their narratives sometimes draw on their wider early experiences in oncology and medicine. Thus, some subthemes may appear less closely tied to CACPs, yet still illuminate how trainees understand these encounters.

The participants’ accounts and the thematic organization reflect this process-oriented perspective, encompassing the trainees’ professional roles and practices, communication skills development, the structured disclosure process, the effects of the consultations on the trainees, and the responses of P&Cs. The five identified themes were as follows: (1) professional role perceptions and oncology practices in cancer progression care, (2) the process of disclosing disease progression, (3) perceptions and reactions of patients and their family caregivers, (4) impacts of disease progression disclosure on medical and nursing trainees, and (5) training and development of communication skills. These themes were addressed by all participants.

### Theme 1: Professional role perceptions and oncology practices in cancer progression care

This first theme highlights how oncology trainees perceive their professional role, emphasizing the combination of scientific expertise and patient-centered engagement. Medical trainees focus on guiding patients through uncertainty and therapeutic decisions, whereas nursing trainees emphasize relational support, emotional presence, and continuity of care. Representative quotations illustrating these points are provided in Table 2. The participants described oncology as a highly complex and multifaceted field that combines scientific rigor with deep human engagement. The discipline’s appeal was linked to the continuous, long-term patient relationships and the interplay among medical, psychological, and social dimensions. The participants emphasized the heterogeneity of cancers, noting that prognosis and therapeutic strategies vary dramatically depending on the tumor type and stage.

**Table 2:** Theme 1: Professional role perceptions and oncology practices in cancer progression care, list of themes, subthemes, and exemplar quotes

**Themes**	**Subthemes**	**Exemplar quotes**
*Main theme 1: Professional role perceptions and oncology practices in cancer progression care*		
Motivations and appeal of oncology		It’s a very complex job—there’s also the psychological side, the nutritional side. And then there’s the scientific aspect. We have so many studies, so many changes happening. It’s also about the relationship with the patient. I find it very different compared to… other jobs where you see the patient for a consultation and then six months later. Here, it’s continuous. (Lucas, oncologist trainee, 5 years of experience)
Specific characteristics of cancers		A thyroid cancer or a testicular cancer—you can treat those quite easily. But pancreatic cancer—within six months, things usually go badly. A glioblastoma in the brain, same thing. The life expectancy is… just catastrophic. (Bianca, oncologist trainee, 4 years of experience)
Diagnostic disclosure and disease progression		And you also have to keep in mind that there’s the announcement of the diagnosis and the announcement of disease progression. The progression announcement is always much easier than the diagnostic one. The diagnostic announcement goes from a moment when the person might not feel sick to ‘I have cancer.’ Whereas a progression announcement goes from: ‘I have cancer under treatment’ to ‘it’s not responding, and we’ll need to change the treatment.’ It’s already much easier for them. So, I dread a progression announcement much less. (Bianca, oncologist trainee, 4 years of experience)
Therapeutic goals		That’s sometimes difficult too—you have to be able to bring up the fact that the goal is no longer to cure, but to relieve symptoms. That’s what the treatment is for at that stage. (Eva, oncologist trainee, 1 year of experience)
Perceived role of the physician regarding disease progression		My role during these consultations is to tell them that the disease is progressing—but that it’s not the end. We still have treatment options, and we’ll need to change the medication, change the chemotherapy or immunotherapy, or even try other methods to treat the tumor effectively, because we haven’t managed to do that yet. In general, my role is really to explain that, to show them that the disease has progressed, and to offer them a way forward. (Marianne, oncologist trainee, 3 years of experience)
Relational dimensions of nursing care	Active listening	For some patients, it’s true that they dare to tell us things more easily. And some of them say, for example, that they have financial worries—and they think, ‘I’m not going to tell the doctor, they don’t care about that, they can’t do anything about it.’ Whereas with us, the nurses, we take the time, and we really ask them about it. (Elise, nurse trainee, 2 years of experience)
	Presence and reassurance	There are quite a few patients who come alone to these visits, so it’s also a way for them to have support—and to have someone who hears what the doctor says, so they can talk about it again afterwards. (Iris, nurse trainee, 1 year of experience)
	Continuity and follow-up of care	We also have to say that we spend more time with the patients—especially when they’re hospitalized — than the doctors do. Doctors are there for the announcements and decisions, but then… we do the rest of the work (laughs). (Diana, nurse trainee, 1 year of experience)

The participants compared diagnostic disclosure with CACP. Diagnostic disclosure emerged as a particularly sensitive moment that was considered emotionally challenging, marking a transition from perceived health to illness. In contrast, progression announcements were often experienced as less distressing because patients were already engaged in treatment. Similarly, according to the participants’ accounts, discussions about therapeutic goals, especially when shifting from curative to palliative intent, required careful communication to balance honesty with hope. Medical trainees perceived their role as guiding patients through uncertainty by explaining the clinical implications, outlining the next therapeutic options, and clarifying how care would continue despite the change.

Nursing trainees highlighted the relational and supportive dimensions of oncology practice in the context of cancer progression. Through active listening, emotional presence, and continuity of care, they provided a stabilizing and empathetic counterpart to the physicians’ technical and decisional roles. Their proximity to patients enabled them to address psychosocial concerns and ensure ongoing support, particularly for those facing the disease alone. While medical trainees primarily described their role as guiding patients through clinical uncertainty and therapeutic decision-making, nursing trainees framed their professional identity around relational continuity, emotional presence, and sustained support across the illness trajectory. These distinct yet complementary role perceptions highlight the interprofessional nature of cancer progression care.

### Theme 2: Process of disclosing disease progression

Participants described disease progression disclosure as a structured, emotionally sensitive process requiring careful preparation, communication, and follow-up. These narratives highlight the coordinated preparation, careful explanation of progression, and provision of emotional support. Representative excerpts are shown in Table 3.

**Table 3:** Theme 2: Process of disclosing disease progression, list of themes, subthemes, and exemplar quotes

**Themes**	**Subthemes**	**Exemplar quotes**
*Main theme 2: Process of disclosing disease progression*		
Emotional and clinical preparation for breaking the news	Anticipation of patients’ and family caregivers’ reactions	Honestly, they’re already in it, they already know. And then they come knowing that we’re going to discuss the results. Because when you start a treatment, you always say, ‘We’ll do chemo, we’ll wait three or six months, then we’ll do a PET-CT or a scan, and then we’ll see.’ So they do the exam, and when they come to see you, they already know it’s to discuss those results. (Lucas, oncologist trainee, 5 years of experience)
	Collective discussion of patient cases during multidisciplinary meetings	We discuss the case beforehand. So if there’s a disease progression, it can happen that this progression is discussed during a tumor board meeting, for example. In that case, we already have more or less an idea of the direction the management will take. (Daniel, oncologist trainee, 5 years of experience)
	Prior knowledge of the diagnosis and management of neutrality	This person [the patient], of course, she sees us—the physician and me—and you can read it on our faces; she already understands that, while waiting for the radiological results, her illness isn’t necessarily very calm. (Iris, nurse trainee, 1 year of experience)
	Medical and logistical preparation	I obviously read the radiologist’s report. I try to know precisely where the progression is and what the possible consequences are. As much as possible, I try to look at the images beforehand. Simply because I think it’s interesting to understand the case well, but also because afterwards the patient asks more technical and precise questions about where the progression is. So I like to already have the answer. I also check their latest blood work. So it’s mainly about mastering all the complementary tests well. (Alex, oncologist, 5 years of experience)
Process and communication strategies	Setting and environment of the disclosure	The ideal environment should be calm, 100% dedicated to the patient. So ideally, there shouldn’t be any phone calls interrupting the consultation—which isn’t always possible. But as much as possible, I try not to answer when it’s a crucial moment for the patient to whom I’m giving the news. (Nathan, oncologist trainee, 3 years of experience)
	Introduction to the disclosure	The patient comes into my office, we say hello, and then they sit down. And well, if it’s bad news, normally I start right away by saying that unfortunately I don’t have good news for them. (Sam, oncologist trainee, 2 years of experience)
		So generally, I always ask the patient how they’re doing. I try to understand a bit what state of mind they’re in before making the announcement. I like to test the waters first and understand how they are, if they’ve already had symptoms before getting to the announcement of the disease progression. (Eva, oncologist trainee, 1 year of experience)
	Explanation of disease progression	I start by explaining things. First, what we have, what we saw on the images or in the context. Or why it looks like there’s a progression. The reason why the current treatment isn’t working. (Bianca, oncologist trainee, 4 years of experience)
	Presentation of a new treatment as a source of hope	From the physician’ side, there are some who give the patient a lot of hope after announcing the progression, but at the same time they’ll almost add, ‘It’s okay, we’ll still have another treatment for you,’ which maybe makes it a bit easier for the patient to accept. (Camille, nurse trainee, 1 year of experience)
	Managing uncertainty regarding prognosis	I think a way that I find relatively pragmatic—but still appropriate—is to say that, in the end, nobody really knows. There are studies for each treatment, and in these studies there’s always a median survival. So, say the median survival is 17 months—that means 50% died before and 50% lived longer. And I find that’s a way to talk about it when people ask, because if they don’t ask, I’m not necessarily going to bring it up. Of course, depending on the disease, I think it’s important to mention it roughly, but not necessarily with exact numbers. (Marianne, oncologist trainee, 3 years of experience)
Support after the disclosure	Physician availability and two-step disclosure	And I always tell patients, if it’s too hard for you, we can stop. If it’s too complicated, we can take a break, see each other again in one or two weeks, and then talk again about a new treatment, if that’s what you want at that time. (Marianne, oncologist trainee, 3 years of experience)
	Follow-up interview conducted by the nurse	I do a follow-up consultation with them to see what they understood, and to answer any questions they might have (...) it’s more about revisiting how they took the news psychologically and emotionally, and what they understood about the new line of treatment—that’s the kind of area we work on afterwards. (Elise, nurse trainee, 2 years of experience)
Participants and modalities of disclosure	Physician’s role in the disclosure	Speaking specifically about announcing disease progression in cancer, those are really tough announcements. So at least where I worked, it was always the doctor who did it. (Nina, nurse trainee, 3 years of experience)
	Disclosure conducted jointly by the trainee physician and senior consultant	It’s true that when you’re a junior resident, if there are really difficult things to announce during these consultations, you’re often paired with one of your superiors. (Charlotte, oncologist trainee, 3 years of experience)
	Disclosure conducted independently by the assistant physician	Every morning, we have an hour of supervision with our senior where we prepare all the day’s consultations, and then we’re on our own during the consultations. If needed, we can call the senior, but generally we’re quite autonomous. (Nathan, oncologist trainee, 3 years of experience)
	Nurse’s presence during the disclosure	In this kind of consultation, our role is really to complement the oncologist’s, and especially to support all the nursing aspects—that means both in the relationship and often in checking whether the patient has understood. (Elise, nurse trainee, 2 years of experience)
Therapeutic relationship and continuity of care	Rotation of trainee physicians	With the rotation of residents and depending on their schedules, sometimes patients see three different doctors over three different appointments. Whereas we nurses—well, there are three of us too in my team—but we always try to assign the same nurse unless there’s an emergency, an absence, or vacations. We try to make sure it’s the same person, and it’s true that the trust-based relationship builds differently than if they keep seeing a different doctor each time. (Anna, nurse trainee, 5 years of experience)
	Prior knowledge or absence of a pre‑existing relationship with the patient	Of course, when you meet someone only two or three times, it’s hard to announce that they have cancer. But I’m not going to cry with them either. Maybe it’s different if it’s a patient you’ve been following for five years, seeing every month, building a relationship with. (Lucas, oncologist trainee, 5 years of experience)
	Nurse–patient relationship	We build connections with these people who are sometimes there for several days, even weeks. So there’s a sort of trust, even a kind of, well—not friendship—but sympathy with them. And since we spend time with them, we’re also kind of forced to open up a bit, to talk about ourselves, about our families. (Camille, nurse trainee, 1 year of experience)
	Impact of disease progression on trust in the relationship	If it goes really badly—which usually it doesn’t—it’s not necessarily because there’s a progression, but because there were already other issues before, in the communication or just in the rapport between two people, like in anything. In my experience, I don’t feel that it [disease progression] impacts the therapeutic alliance. (Alex, oncologist trainee, 5 years of experience)

Before meeting the patient, the participants reported coordinating with colleagues during multidisciplinary meetings and preparing by reviewing medical data and anticipating patients’ and family caregivers’ reactions. Physician trainees emphasized the importance of being fully informed about the case and mastering the technical aspects of the results, whereas nurse trainees noted that patients often sensed the seriousness of the situation even before the consultation began. Nursing trainees described a distinct temporal positioning in the disclosure process. While physicians focused on preparing and delivering medical information during the consultation, nurses often engaged with patients before and after the announcement, addressing anticipatory anxiety, clarifying understanding, and providing emotional containment.

The participants highlighted the need for an appropriate setting for the disclosure, ensuring calm and privacy. Consultations could take place in the physician’s office or, for hospitalized patients, at the bedside. The introduction to the discussion varied by professional, with some preferring a direct approach and others beginning by assessing the patient’s emotional state. Explanations of disease progression were often accompanied by the presentation of new therapeutic options, helping physician trainees facilitate a sense of hope. Physician trainees also described strategies for addressing uncertainty, such as using general references to survival statistics without providing precise figures unless requested.

Providing support to patients after the disclosure was described as an essential component of the process. Physician trainees frequently offered follow-up appointments to give patients time to process the information, while nurses conducted additional interviews to assess understanding and provide emotional support.

The disclosure meeting generally involved the physician, sometimes accompanied by a resident, a nurse, or a family caregiver. The primary responsibility for announcing progression rested with the physician, though junior physicians often worked alongside their supervisors during complex consultations. Given that the interviewees were still in training and had limited experience, they most often attended CACPs as observers or as support to a more senior physician. The nurses described their presence as complementary, focusing on patient comprehension and emotional reassurance.

In describing the continuity of care, the participants highlighted the effects of staff rotation and varying levels of familiarity with patients. On the one hand, trainees reported that frequent assignment changes could result in patients seeing different physicians at each consultation. Nurse trainees, on the other hand, often followed the same patients throughout their treatment and hospitalization, enabling them to build relationships of trust and mutual understanding. Some explained that these relationships developed naturally over time and through shared conversations, while others noted that announcing disease progression to a long-term patient could be particularly emotional.

### Theme 3: Perceptions and reactions of patients and their family caregivers

Patients and family caregivers displayed diverse emotional and decision-making responses, reflecting the complexity of navigating cancer progression. Family involvement emerged as a central element in providing emotional support and aiding decision-making. Individual coping styles shaped responses, highlighting the need for flexible, patient-centered communication. Representative quotations illustrating these experiences are presented in Table 4.

**Table 4:** Theme 3: Perceptions and reactions of patients and their family caregivers, list of themes, subthemes, and exemplar quotes

**Themes**	**Subthemes**	**Exemplar quotes**
*Main theme 3: Perceptions and reactions of patients and their family caregivers*		
Support and mediation from family caregivers	Emotional support	It can be supportive because—I mean, for the person, it’s also good to have someone by their side who supports them through these challenges. I think that’s important. (Iris, nurse trainee, 1 year of experience)
	Informational role and facilitation of understanding	There’s a lot of information at the beginning, so having another person to listen for them is good because they’re sure they won’t forget anything. (Simone, nurse trainee, 1 year of experience)
Emotional reactions to the announcement	Anticipation and psychological preparation	It depends on the patients (laughs), because those who react well… well, some patients expect it when they have more symptoms and then feel worse. (Iris, nurse trainee, 1 year of experience)
	Emotional shock	Even with that state of shock, because it was still something violent, and she was going through a period of emotional upheaval, but still maintaining a bit of control over the situation. (Nina, nurse trainee, 3 years of experience)
	Relief or acceptance	Yes, but there’s something quite surprising behind it. And she expressed it well: ‘I’m relieved to know.’ (Iris, nurse trainee, 1 year of experience)
	Hiding their emotions	Sometimes it’s true that patients might react in a way to save face in front of the doctors, or maybe they don’t fully realize it. (Simone, nurse trainee, 1 year pf experience)
	Sadness and disappointment	There are indeed moments when they might cry or express sadness, or maybe not anger, but more… disappointment rather than anger. (Anna, nurse trainee, 5 years of experience)
	Anger and revolt	So there’s anger too. Like, it’s been a while since they told me about this appointment, the announcement was two months ago, and I only got your appointment now—what’s going on? So there’s anger against the system, against… the illness isn’t highlighted, but it’s mainly about the care process. That can be really intense. (Livia, nurse trainee, 5 years of experience)
Use of complementary approaches		We also always offer them complementary therapies if they want. It can be hypnosis, art therapy, or seeing a psychologist. These are things we propose all the time, and we tend to suggest them more when the emotional load is quite heavy. So we’re oncologists, but we don’t work alone. It’s also important to include other specialists. (Sam, oncologist trainee, 2 years of experience)
Decision-making processes and patient autonomy	Allowing the patient to choose	So I always try to give them the choice, to show them the options, and to let them know that I’m here to help. (Bianca, oncologist trainee, 4 years of experience)
	Providing the necessary information for decision-making	It was an interesting situation because there’s no right or wrong. The only thing we have to be careful about is giving the patient all the information they need to be safe. Once that’s done, they’re free to do what they want. (Daniel, oncologist trainee, 5 years of experience)
	Listing therapeutic options	And I like to say, ‘There are other possible paths.’ But we can also say, at the extreme, ‘If you don’t want to, we could also do nothing. But of course, I won’t abandon you—if there are symptoms, we’ll manage them, and we’ll proceed step by step.’ I like to be a bit provocative like that because we know we could treat, but the treatments can be very toxic. Then, if the patient wants to discuss it, we talk about the pros and cons. (Nathan, oncologist trainee, 3 years of experience)
	Delegating the decision to the doctor	Clearly, giving them the choice also puts pressure on them, because otherwise, it’s the doctor who decides. And sometimes, some say, ‘I don’t know, it’s up to you to decide,’ while others are fully clear and make decisions. (Lucas, oncologist trainee, 5 years of experience)

Family caregivers were frequently highlighted as crucial sources of support, providing emotional reassurance and practical assistance in understanding medical information. Patients’ reactions to medical announcements were diverse, ranging from anticipation and psychological preparation to shock, relief, emotional concealment, sadness, and anger. The participants reported that these reactions were often shaped by individual coping styles, prior expectations, and perceived quality of communication with HCPs.

Participants also discussed the integration of complementary approaches, such as psychological support, hypnosis, or art therapy, particularly when the emotional burden was high. Decision‑making processes varied considerably, encompassing active patient choice, informed deliberation of treatment options, and, in some cases, delegation of decisions to the physician. Participants emphasized the importance of providing comprehensive information while respecting patient autonomy, balancing guidance with support. Overall, participants highlighted the centrality of family involvement, emotional attunement, and flexible, patient-centered decision-making in navigating the clinical experience.

### Theme 4: Impacts of disease progression disclosure on medical and nursing trainees

Disclosure of disease progression had multifaceted impacts on trainees, including emotional strain, professional challenges, and the development of coping strategies. Illustrative quotations supporting these points are provided in Table 5.

**Table 5:** Theme 4: Impacts of disease progression disclosure on healthcare professionals, list of themes, subthemes, and exemplar quotes

**Themes**	**Subthemes**	**Exemplar quotes**
*Main theme 4: Impacts of disease progression disclosure on healthcare professionals*		
Emotional reactions regarding cancer progression disclosure	Fatigue and loss of energy	In general, my emotional state is quite neutral, and it can happen that sometimes, when I am a bit more emotionally involved, I feel somewhat drained of energy. (Claire, oncologist trainee, 2 years of experience)
	Sadness mitigated by experience	It’s always still a sad and unpleasant situation for the doctor. For me, it affects me less because I’ve done it often. Unfortunately, over time, it affects you a bit less. Still sad, but you’re less impacted. (Alex, oncologist trainee, 5 years of experience)
	Emotions depending on the patient’s reaction	It really depends on the patient’s reaction (...) If the patient “breaks down,” shows emotions, cries, seems desperate, that’s when it’s hard. I can also feel a bit of the same, experiencing emotions myself, even sometimes to the point of tears. (Eva, oncologist trainee, 1 year of experience)
	Professional frustration	There are cases where we sometimes feel like we are chasing a disease that slips away, that we can never fully control. That’s when frustration sets in. (Daniel, oncologist trainee, 5 years of experience)
	Stress and anticipatory anxiety	Honestly, it stresses me out, makes me anxious, a bit anxious, fearful, like not knowing what to say, not being adequate. (Elise, oncologist trainee, 2 years of experience)
	Evolution of emotional experience with time	It was mostly at the beginning of my training, and the more I progress, the less I manage to detach, I think it’s a little… At the start, I was completely without emotions. (Bianca, oncologist trainee, 4 years of experience)
	Feelings of guilt	So there’s a bit of guilt when the patient breaks down. I tell myself, I’m guilty—I’m responsible for having given this to them, or for having done that. (Marianne, oncologist trainee, 3 years of experience)
	Repercussions on private life	I really think that if I’ve done things correctly or I’m aligned with it, that’s fine. But if I have the slightest doubt, or something I didn’t discuss with a senior when I should have, or I think, “Oh, I should have done this with the patient, I didn’t think of it,” that’s when it can follow me home. (Sam, oncologist trainee, 2 years of experience)
Professional constraints and challenges	Lack of specific experience in oncology	So I found it difficult, because it was really about discussing everything with the senior. Essentially, the senior told me what I had to say, and then I relay it to the patient alone. Sometimes the patient has unexpected questions. And it’s true that in breaking bad news, it’s a bit tricky to say, “I don’t know.” (Eva, oncologist trainee, 1 year of experience)
	Repeated announcements	The situations that affect us more, or an accumulation of… sometimes we have waves of progressions all at once, and that definitely stays on your mind at home. (Bianca, oncologist trainee, 4 years of experience)
	Lack of time	It’s true that we have a very full schedule and consultations every thirty minutes. But, except in very specific situations, we rarely spend more than thirty minutes, even for delivering bad news. Ideally, I would like to have an hour. (Daniel, oncologist trainee, 5 years of experience)
	Organizational and institutional constraints	I’d like more flexibility sometimes! It’s… these are small things, but a mother who wants her treatment at 10 or 11:30 because she dropped off her kids, did three errands, and needs to pick them up at noon. Sometimes it doesn’t work because we don’t have enough slots. This was already a problem in clinics or hospitals. (Diana, nurse trainee, 1 year of experience)
	Nurses compensating for communication gaps	With the head doctor, it’s a bit more complicated because, over time, as she’s immersed in her work, sometimes we find her comments a bit inappropriate, and we’ve even had feedback from patients telling us that (...) and sometimes she repeats exactly what the assistant doctor just said, and sometimes she goes off into incoherent statements, and we have to fix it afterward. (Anna, nurse trainee, 5 years of experience)
	Emotional identification with the patient	It affects me, especially with patients I can identify with. So patients my age… who have families, young children—that really affects me. (Daniel, oncologist trainee, 5 years of experience)
	Harmonizing interprofessional communication	We know that it’s important for all doctors involved to be really (…) aware of what we’re going to say to the patient, and that everyone talks about the same thing, proposing the same approach. Not that the oncologist says one thing and the gynecologist, unaware, says the opposite—because that is very unsettling. Even small things that may seem unimportant to us can be very destabilizing for a patient. So consistency in communication is key. (Bianca, oncologist trainee, 4 years of experience)
Coping strategies	Drawing on accumulated experience	If I look at myself in my first year versus my last year, I feel more ready to welcome patients’ emotions. But I don’t think it’s because I have more specific tools or learned theory, or the “right way” to do things, but more because of the stance you adopt—you have more knowledge, more experience, it’s kind of the end result. (Nina, nurse trainee, 3 years of experience)
	Maintaining emotional distance	If I didn’t differentiate between work and personal life, it would be too hard. So, I think you’re forced to put up a kind of shell or distance yourself from what happens at work. It would be unbearable, and I think you don’t really have a choice. (Anna, nurse trainee, 5 years of experience)
	Reassurance from available treatments	But I try not to move on too quickly, because it’s a relief for us to say there’s still another line of treatment. But I try not to jump on that opportunity to downplay the progression. I want it to be acknowledged and understood as such. (Charlotte, oncologist trainee, 3 years of experience)
	Offering concrete solutions to patients	I also work closely with psycho-oncologists, which is very important. Already, when we have a first diagnosis, I always proactively discuss it. We also have a folder we always give the patient with all the data and phone numbers, and in situations with changes, I almost always revisit the psycho-oncologist topic. (Bianca, oncologist trainee, 4 years of experience)
Available supports	Seeking support from one’s supervisor	If I feel stress rising, if I lose control, I know I can say, “Listen, I suggest we… just take a pause and have the doctor join us so we can discuss together.” We are part of the same team; we work as a team, and I think it’s good to do that. (Sam, oncologist trainee, 2 years of experience)
	Debriefing with colleagues	Talking to colleagues afterward, because you’re not alone in oncology—you have senior doctors, colleagues who are assistant doctors, nurses. You can always talk and say, “I had to deliver this news, the patient reacted badly, it was hard.” I think talking to other colleagues is good to move on a bit. (Marianne, oncologist trainee, 3 years of experience)
	Sharing with relatives	My partner is also very open to that, and I know that on difficult days, I can debrief with him at the start of the evening. (Camille, nurse trainee, 1 year of experience)
	Psychological support	I started psychological follow-up last year in July, and I don’t know if it particularly helps me, but I think it was necessary. (Marianne, oncologist trainee, 3 years of experience)

Participants reported diverse emotional reactions and personal adjustments, including fatigue and emotional exhaustion after consultations, particularly when the interaction was intense or when they felt closely connected to the patient. Sadness was a common reaction, though many noted that repeated exposure and clinical experience helped them develop a certain emotional distance over time. Others described their emotions as strongly influenced by the patient’s reaction; moments when patients cried or expressed despair were perceived as particularly difficult. Feelings of frustration, anxiety, and guilt were also reported. Some participants noted that these experiences occasionally affected their private lives, while others emphasized their ability to separate professional and personal spheres and manage emotions through detachment or compartmentalization. Although both medical and nursing trainees reported emotional strain, nursing trainees more frequently described distress related to prolonged emotional involvement and repeated exposure to patients’ suffering over time, particularly when strong bonds had been established.

The participants also referred to various professional constraints and challenges associated with disclosure. Less experienced physicians often cited a lack of specific training and difficulty managing unexpected questions. Time constraints and institutional pressures limited the opportunity to provide adequate emotional support during consultations. Nurse trainees sometimes described compensating for communication lapses by physicians, while both nurse and physician trainees emphasized the importance of consistent interprofessional communication to avoid giving patients contradictory messages. Emotional identification with patients of similar ages or life circumstances was also described as a significant source of distress.

Participants relied on several strategies to cope with these challenges. Experience was often considered a protective factor, helping to reduce emotional intensity and increase confidence. Maintaining emotional distance was perceived as necessary for self-preservation. Some participants found reassurance in the availability of further treatment options or in concrete solutions, such as psychological support for patients. Positive feedback from P&Cs also served as an important source of motivation and validation.

Finally, the participants mentioned a range of support mechanisms within and outside the workplace. Many emphasized the value of discussing difficult consultations with supervisors and colleagues as a form of informal debriefing. Sharing experiences with relatives or partners also helped release tension. Some medical trainees reported seeking psychological support when emotional strain accumulated, recognizing it as a necessary resource in sustaining their professional well-being.

### Theme 5: Training and development of communication skills

Trainees highlighted multiple approaches to acquiring communication skills, balancing structured protocols with experiential learning. Core skills such as empathy, clarity, and adaptability were consistently emphasized, yet participants reported gaps in formal training and challenges in applying protocols authentically. Representative quotations are shown in Table 6.

**Table 6:** Theme 5: Training and development of communication skills, list of themes, subthemes, and exemplar quotes

**Themes**	**Subthemes**	**Exemplar quotes**
*Main theme 5: Training and development of communication skills*		
Training modalities	Theoretical training	I can try to find the algorithm later. I think it’s SPICES… Anyway, no, it was SPIKES. There’s something called SPIKES. So, we used that quite a lot, and I think it was quite useful. It’s something they also teach in medical school. It’s a series of steps for how to handle the situation—you set up the interview, you explain the purpose, like ‘We’re going to discuss your results.’ Then, typically, the second step is to ask the patient what they’ve understood, what they expect to hear. And then there are several steps that lead to breaking the news properly. I apply it, honestly. I think it’s a good method—it’s systematic, and it helps you not forget anything. (Marianne, oncologist trainee, 3 years of experience)
	Role-playing and simulated patients	At school, we have what we call simulated patients—real people like you and me, who come in, and who have some theater training or are volunteers, I’m not sure exactly. Basically, they play a role—a heart attack, a decompensation, borderline personality, whatever, they’re multi-purpose, it’s great (laughs). And then, for example, it’s a patient who plays the role of someone who’s just been told the news. The doctor has just broken the news, and then we come into the hospital room afterward—the patient starts crying, explains that they’re devastated, etc. And we have to learn how to react in front of this patient. (Daniel, oncologist trainee, 5 years of experience)
	Role models	We also learn a lot just by being next to others and watching our supervisors or other people do it. (Alex, oncologist trainee, 5 years of experience)
	Field experience and trial-and-error learning	Independently, we also learn through making mistakes. Yes, by realizing that sometimes there are things we should have communicated differently. From a situation that doesn’t go as well as we’d hoped—maybe the communication wasn’t right. A lot of it is learned through practice, really. (Eva, oncologist trainee, 1 year of experience)
	Mentorship and direct supervision	We often have to deliver bad news, let’s say, in the presence of a senior or a supervisor. So you could say it’s a kind of training, because afterward we get feedback on what we did. (Nathan, oncologist trainee, 3 years of experience)
Communication skills	Clear and appropriate vocabulary	For me, it’s really important to use words that patients can understand—not necessarily overly medical language. For instance, the word metastasis is fairly common, and most people know what it means, but if they don’t, I explain it. (Bianca, oncologist trainee, 4 years of experience)
	Empathy and compassionate listening	I also think it’s important to show empathy, but to try to stay more or less neutral. Maybe if you get too emotional, patients pick up on that and feel like… it’s the end of the world. (Eva, oncologist trainee, 1 year of experience)
	Honesty and transparency	You should never lie. Personally, I never lie. I never try to sugarcoat things because I think patients prefer it when we’re honest. (Marianne, oncologist trainee, 3 years of experience)
	Adaptation to the patient	I’ve tried several ways, and I’m not sure any one of them really suits me. It always depends on the moment—on the patient, on what they’ve understood about their situation. (Sam, oncologist trainee, 2 years of experience)
	Use of silence	So we gave her quite a bit of time—moments of silence—and time for her to express what she was feeling as best as she could. (Iris, nurse trainee, 3 years of experience)
	Nonverbal communication and physical contact	That’s important too—all the nonverbal techniques, like if you see the patient is okay with it, you can touch their hand or place your hand on their shoulder. For some people that feels very natural, but others are less tactile, not very talkative—you have to feel it out. (Livia, nurse trainee, 6 years of experience)
	Influence of the healthcare professional’s personality	I think it’s about the doctor’s personality. Honestly, some doctors are tough, cold. They’re just not empathetic. I don’t know what they were like when they were younger, but it’s often the senior doctors. (…) I get the impression they’re cold in every aspect of life. Even when you have lunch with them, they’re rigid, socially distant sometimes. I think it’s more of a personality trait that may worsen with time, but it was already there. I don’t think someone who’s truly empathetic can become completely unempathetic at the end of their career. Maybe they’re affected less, but they still remain somewhat empathetic. (Simone, nurse trainee, 1 year of experience)
Limits of training	Optional nature of training	What I find unfortunate is that this kind of training isn’t mandatory in oncology medical education—even though we have to deliver bad news every week. (Elise, nurse trainee, 2 years of experience)
	Protocol paradox	We had quite a few courses on this, and we did simulations. There was sort of a procedure—steps to follow—but maybe that’s why it becomes a bit robotic at first. We tend to want to follow the checklist, but it doesn’t always work. (Sam, oncologist trainee, 2 years of experience)
	Insufficient or absent training	But me, no—I didn’t have any training. We have some psycho-oncology meetings where we discuss difficult situations. And as young doctors, we really wanted that kind of training. Because those are clearly the hardest moments in this job. (Charlotte, oncologist trainee, 3 years of experience)
Hierarchical relationships and supervised learning	Collaborative hierarchy in oncology	The medical world is indeed very hierarchical. But that’s normal—that’s how it works. Still, in oncology, it feels a bit more like a family, and I think that really helps. (Charlotte, oncologist trainee, 3 years of experience)
	Tensions or disagreements with a superior	At one point, I wanted to tell her, ‘Just say it—tell them the truth.’ It’s not even that we hide things, but it’s that oncologists are so afraid to say a patient is going to die (laughs). And later, with that same oncologist, we went to the patient’s room—she wanted to keep doing things, and I was so upset that it’s one of the only times I was a bit firm. I said, ‘No, we have to let him be—he’s in palliative care now, we need to stop.’ I think that’s the only time I really went against a senior doctor. (Sam, oncologist trainee, 2 years of experience)

Medical and nursing trainees described multiple training modalities, ranging from theoretical instruction, often structured around communication frameworks such as the SPIKES (Setting, Perception, Invitation, Knowledge, Emotions/empathy, and Strategy/summary) protocol [5], widely adopted in medical curricula to support the delivery of unfavorable news, to experiential learning through role-playing with simulated patients. Observation of senior colleagues and mentorship during clinical practice also played a key role, allowing learners to internalize effective communication strategies and receive real-time feedback. In addition to structured teaching, participants underscored the importance of learning through practice, including moments of trial and error that foster professional growth and self-reflection.

The participants identified a set of core communication skills, including clarity, honesty, empathy, and the ability to tailor their approach to each patient’s emotional and cognitive state. Nonverbal cues, silence, and appropriate physical contact were recognized as integral to establishing trust and conveying compassion. The influence of individual personality traits was also acknowledged to shape communication styles and the degree of emotional engagement.

Despite these efforts, participants noted significant limitations in training, particularly its optional or insufficient nature in oncology education. Although communication protocols were reported to provide useful structure, some participants perceived them as overly mechanical, highlighting a “protocol paradox” that can hinder authentic connection and adaptability.

The participants described oncology as a field with strong hierarchical structures that, in their experience, are often supportive. Junior physicians often delivered difficult news under the supervision of senior colleagues, who provided immediate feedback and assistance when consultations became challenging. Some participants emphasized the reassuring presence and availability of their superiors, whereas others mentioned moments of disagreement or hesitation to speak up due to hierarchical norms.

## Discussion and conclusion

### Discussion

This study provides an in-depth exploration of how medical and nursing trainees experience consultations involving treatment changes due to cancer progression. Our findings underscore the multidimensional nature of these interactions, in which trainees must navigate clinical accuracy, emotional responsiveness, and relational complexity simultaneously. Disclosing cancer progression is not merely the transmission of medical facts but a carefully orchestrated process that involves preparing the patient, communicating clearly and empathetically, and supporting both understanding and emotional adjustment. Trainees’ experiences reveal the need to balance realism with hope, manage their own emotional responses, and coordinate effectively with colleagues, patients, and family caregivers.

Although our participants perceived the disclosure of disease progression as less emotionally impactful than the disclosure of the initial diagnosis, disclosing progression remains challenging because it combines the clinical complexity associated with adapting treatment with emotional management. The participants reported that, although patients often anticipate these consultations, the announcements remain emotionally intense and affect trainees’ well‑being. Our results extend previous findings on the importance of preparation, relational knowledge, and follow-up support [4, 13, 32].

In contrast to the study by Kolly et al. [13], which explored the perspectives of experienced oncologists and nurses in CACPs, the present findings reflect the standpoint of trainees. Kolly et al. [13] reported that HCPs gradually develop confidence and emotional distance through repeated exposure to CACPs,however, the participants in our study described a more uncertain and affectively charged experience. Emotional impacts reported by the participants included anxiety, sadness, frustration, guilt, and a pervasive sense of inadequacy, consistent with the literature on trainees [6, 15, 33]. Our participants’ accounts of emotional vulnerability echo findings that inexperienced trainees often struggle with confidence and are strongly affected by patients’ reactions [18, 34]. In our study, however, these experiences emerged in the context of a specific and, to our knowledge, previously unexamined moment in the care trajectory: the announcement of a treatment change within the CACP setting. Although occasional positive emotions, such as gratitude or satisfaction in helping patients, have been noted in some studies [16, 18], our results indicate that these emotions remain secondary to the dominant emotional strain associated with breaking bad news. Importantly, our findings highlight the unique contribution of nursing trainees in cancer progression consultations. Their emphasis on relational continuity, emotional availability, and patient comprehension complements the physicians’ focus on diagnostic and therapeutic decision-making. This interprofessional dynamic appears particularly salient in the context of disease progression, where patients often require sustained emotional support beyond the moment of medical disclosure.

Our participants all had prior experience in delivering bad news and did not report feeling incapable of doing so, which aligns with studies showing that trainees with previous exposure feel more capable and less apprehensive than those without such experience [15, 33]. In our study, the participants described relying mainly on peer dialogue, informal debriefing, and occasional support from senior HCPs, as well as personal strategies such as reflection or temporary distancing. The literature similarly indicates that HCPs in training with prior experience tend to use more adaptive coping strategies, seeking support from close others or experienced HCPs and engaging in restorative activities,in contrast, inexperienced trainees more often report non-adaptive or avoidant responses [33]. A more distinctive element in our study is the strong importance participants placed on being honest and transparent with patients about the implications of the treatment change. Several participants explicitly mentioned their effort not to minimize or obscure the situation. This emphasis may reflect broader evolutions in professional training, where emotional literacy, open communication, and ethical clarity are increasingly promoted. Thus, trainees may be more comfortable acknowledging and sharing emotions, along with being more attentive to providing clear and forthright information.

Together, these parallels suggest that supportive relationships and accessible guidance are central to how trainees manage the emotional challenges of breaking bad news [18, 35]. Furthermore, the attitudes observed in our participants indicate generational shifts in openness, transparency, and the integration of emotional competencies into clinical practice.

The participants emphasized insufficient formal training and relied on clinical exposure and senior HCP observation, a finding that echoes longstanding concerns in the literature regarding inadequacies in training for breaking bad news [17]. This finding mirrors consistent evidence that observing experienced HCPs deliver bad news helps trainees develop their own approach, while personally delivering bad news remains a pedagogically impactful experience and is associated with greater confidence and lower stress [15, 35, 36]. The sense of inadequate preparation reported by our participants reflects wider concerns documented in the literature, in which many students have been found to consider their training insufficient or poorly timed, often relying on theoretical methods [37–39]. Consistent with our findings, previous studies have also underlined the value of experiential learning, peer dialogue, and supervised practice, with simulation identified as both challenging and highly formative [18, 34].

An important point emerging from our data is the strong willingness among trainees to access supervision and structured support, in contrast with reports from clinical settings indicating that senior oncologists rarely attend optional supervision sessions. This generational difference suggests that the early training period constitutes a critical window for providing guidance. During these years, clinicians are still developing their routines and remain flexible enough to integrate new communicational and emotional skills, and their “still in training” status legitimizes help-seeking. However, later in the professional trajectory, routines tend to be firmly established, and seeking support may be perceived as a sign of weakness in the highly demanding medical environment [13, 32]. This finding underscores the importance of ensuring that supportive structures and supervision are accessible and encouraged during this formative phase.

The SPIKES protocol, a six-step communication framework designed to guide clinicians through the structured delivery of bad news, was described as a helpful reference; however, the participants stressed flexible, context-sensitive use, consistent with research indicating that strict adherence may not meet patients’ individual needs [40–42]. This finding aligns with the broader literature showing that SPIKES is one of the most widely used communication protocols in medical training [37] and that its structured format can improve students’ satisfaction, knowledge, and performance when taught through active, experiential methods [43, 44]. However, consistent with our participants’ emphasis on adaptation, several studies highlight that the strict or mechanical application of SPIKES may not meet patients’ needs: perceived adherence is often incomplete, especially in exploring patient understanding, eliciting preferences, and collaboratively planning next steps [41]. Furthermore, research has demonstrated that patients’ expectations and preferences vary widely across psychological, clinical, and sociodemographic factors [40, 42], underscoring the need for a nuanced, individualized approach to the protocol rather than a step-by-step formula [45].

From a medical education perspective, these findings underscore the importance of interprofessional training approaches that explicitly acknowledge and value nursing trainees’ roles in communication, emotional care, and continuity. Recognizing these complementary competencies may not only improve patient care but also enhance mutual understanding and collaboration among trainees from different professional backgrounds. From a nursing education perspective, these findings highlight the importance of training nursing trainees in relational continuity, emotional support, and patient comprehension during cancer progression care. Unlike medical trainees, nurses are often involved before and after the disclosure, placing them in a key position to address psychosocial needs and emotional adjustment. These results underscore the need to explicitly recognize and integrate these competencies into oncology nursing education and interprofessional training.

Finally, our results suggest that choosing oncology as a specialty and developing a professional identity are dynamic processes shaped by patient care experiences, emotional challenges, and available institutional support. The participants highlighted the appeal of oncology due to long‑term patient relationships, multidisciplinary practice, and meaningful care, findings consistent with those of Faivre et al. [14] and Julius and McCarthy [18]. The participants also noted that disclosing disease progression, while emotionally demanding, contributes to their professional growth by strengthening their sense of competence and ethical responsibility, in line with studies showing that difficult clinical encounters play a key role in identity formation and emotional resilience [6, 16, 46].

### Limitations

This study has several limitations that should be considered. First, the study was conducted exclusively in public hospitals in Switzerland, limiting the generalizability of the findings to other healthcare systems, cultures, or institutional contexts. Medical practices, including communication training, can differ substantially across countries and even within the same country [15, 18, 38, 47]. For example, the timing and nature of communication training in medical curricula vary widely, with theoretical courses typically dominating the first years and practical clinical training, including communication skills, introduced later [37, 40].

Second, the participant sample was predominantly female, particularly among nurses (100%). This gender imbalance may have influenced the experiences and communication strategies reported, limiting the applicability of the findings to a more gender-balanced population, including male HCPs who might perceive and respond to patient interactions differently. Had the sample included more men, we might have observed comparatively less verbal empathy or fewer expressions of emotional vulnerability, given evidence that female physicians tend to self‑report greater empathic concern than male colleagues (although behavioral empathy measures vary) [48]. This raises the question of whether the intense relational and emotional engagement described by our predominantly female “young” participants reflects a generational shift, a gender-specific tendency, or their intersection.

Finally, several sources of potential bias should be acknowledged. Recruitment and self‑selection biases may have favored participants who are more comfortable with delivering bad news or more motivated to share their experiences, potentially limiting the diversity of perspectives. For example, our team tried to conduct this study in France; however, no medical trainees agreed to be interviewed. In addition, the use of snowball sampling may have resulted in a participant pool in which trainees knew each other or worked within the same unit or micro-culture. This could have influenced the range of experiences and perspectives reported, potentially over-representing certain communication practices or cultural norms specific to these settings. Additionally, social desirability bias may have influenced participants’ responses, leading some to emphasize their professional competence rather than discuss difficulties or errors. As noted in our previous study [13], this focus on performance is inherent to medical culture, where expressing emotions or reactions to challenges is often considered inappropriate [49], contributing to a culture of invulnerability [32].

### Practice implications

The present study highlights several practical implications for improving communication training in healthcare, particularly in oncology. First, there is a clear need to integrate communication training more systematically and to incorporate it as a mandatory component of medical and nursing curricula. Evidence shows that experiential learning methods, such as role-playing, simulated patients, and supervised clinical interactions, are highly valued by students and significantly improve self-efficacy, empathy, and communication skills [36, 38, 39, 50]. Students often prefer active, skill-based learning over passive methods, highlighting the importance of structured opportunities to practice delivering bad news [46]. However, pedagogical approaches vary widely across and within countries, suggesting the need for locally adapted yet standardized frameworks for communication training [15, 18, 37, 38, 47].

Second, communication training should be tailored to specific clinical scenarios, such as disclosing cancer progression, which requires balancing the delivery of bad news with maintaining patient hope through information about potential treatment options [13]. Flexibility in applying structured protocols such as SPIKES is recommended, with emphasis on adapting communication to the patient’s emotional state and needs rather than strict adherence to formal steps [13, 38, 51].

Finally, improving access to support resources is essential for preparing HCPs to manage the emotional burden of disclosing unfavorable medical information. Structured debriefings, mentorship programs, and the availability of psychologists can help students and professionals navigate the psychological stress inherent in delivering bad news [33, 35, 38, 52]. Research has shown that integrating emotional management training alongside communication training enhances performance, self-confidence, and resilience, while fostering a culture of reflection and interprofessional collaboration [33, 46].

### Conclusion

This study explored the experiences of medical and nursing trainees in delivering bad news about cancer progression within Swiss public hospitals. We highlighted how the participants perceived and navigated these challenging situations, noting the strategies they used to communicate with clarity, empathy, and professionalism. The findings showed that while HCPs strive to balance honesty and support, they face difficulties such as limited structured support, a need for mandatory communication training, and reliance on informal peer support to cope with the emotional burden.

Overall, the study provides insight into the nuances of delivering news of cancer progression in clinical practice and emphasizes the importance of adaptive, context-sensitive communication strategies. These results highlight actionable areas for improvement, including enhancing training, providing structured support, and fostering team-based approaches to better equip HCPs for these demanding interactions.

## Data Availability

The datasets generated and/or analyzed during the current study are not publicly available due to the sensitive nature of the interview data and the risk of compromising participant confidentiality. However, the corresponding author may make the data available upon reasonable request and subject to ethical approval.
